# Head-to-Head Comparison between Peptide-Based Radiopharmaceutical for PET and SPECT in the Evaluation of Neuroendocrine Tumors: A Systematic Review

**DOI:** 10.3390/cimb44110373

**Published:** 2022-11-07

**Authors:** Giulia Poletto, Diego Cecchin, Stefania Sperti, Luca Filippi, Nicola Realdon, Laura Evangelista

**Affiliations:** 1 Nuclear Medicine Unit, Department of Medicine DIMED, University of Padua, 35128 Padua, Italy; 2 Department of Nuclear Medicine, Santa Maria Goretti Hospital, 04100 Latina, Italy; 3 Department of Pharmaceutical and Pharmacological Sciences, University of Padua, 35131 Padua, Italy

**Keywords:** 68Ga-DOTA(0)-Phe(1)-Tyr(3)-octreotide, gallium Ga 68 DOTATATE, 68Ga-DOTANOC, 99mTc-EDDA/HYNIC-TOC, 111In-DTPA(0)-octreotide, SPECT, PET

## Abstract

We compared head-to-head the most used radiolabeled peptides for single photon computed emission tomography (SPECT) and positron emission tomography (PET) imaging of neuroendocrine tumors (NETs). A comprehensive literature search was performed in PubMed, Web of Science, and Scopus databases. The following words, coupled two by two, were used: ^68^Ga-DOTATOC; ^68^Ga-DOTATATE; ^68^Ga-DOTANOC; ^99m^Tc-EDDA/HYNIC-TOC; ^64^Cu-DOTATATE; and ^111^In-DTPA-octreotide. Moreover, a second-step search strategy was adopted by using the following combined terms: “Somatostatin receptor imaging,”; “Somatostatin receptor imaging” and “Functional,”; “Somatostatin receptor imaging” and “SPECT,”; and “Somatostatin receptor imaging” and “PET”. Eligible criteria were: (1) original articles focusing on the clinical application of the radiopharmaceutical agents in NETs; (2) original articles in the English language; (3) comparative studies (head-to-head comparative or matched-paired studies). Editorials, letters to the editor, reviews, pictorial essays, clinical cases, or opinions were excluded. A total of 1077 articles were found in the three electronic databases. The full texts of 104 articles were assessed for eligibility. Nineteen articles were finally included. Most articles focused on the comparison between ^111^In-DTPA-Octreotide and ^68^Ga-DOTATOC/TATE. Few papers compared ^64^Cu-DOTATATE and ^68^Ga-DOTATOC/TATE, or SPECT tracers. The rates of true positivity were 63.7%, 58.5%, 78.4% and 82.4%, respectively, for ^111^In-DTPA-Octreotide, ^99m^Tc-EDDA/HYNIC-TOC, ^68^Ga-DOTATATE/TOC and ^64^Cu-DOTATATE. In conclusion, as highly expected, PET tracers are more suitable for the in vivo identification of NETs. Indeed, in comparative studies, they demonstrated a higher true positive rate than SPECT agents.

## 1. Introduction

Neuroendocrine tumors (NETs) are a heterogeneous family of relatively uncommon neoplasms originating from endocrine cells. They can originate from the lung, thymus, gastrointestinal tract, or pancreas, sharing some morphological and immunohistochemical characteristics. In recent years the incidence of NETs has increased, although it is still considered a rare neoplasm [1,2].

More than 80–90% of NETs express somatostatin receptors (SSTR), which are integral membrane glycoproteins that can be physiologically found in different tissues throughout the body, such as the spleen, kidneys, liver, pituitary, thyroid, and adrenal glands. Five different types of SSTR (sst1–sst5) have been identified with different tissue distributions [1,2]. The somatostatin receptor type 2 (sst2) is the one expressed more frequently by NETs, but also, sst3 and sst5 can be significantly found. The expression of SSTR by NETs offers a very specific target for diagnostic imaging and therapy. Thus, techniques such as single photon emission tomography (SPECT) and positron emission tomography (PET) can be used for the detection of NETs combined with conventional imaging modalities such as computed tomography (CT), magnetic resonance (MR), and ultrasound [2].

In 1989, the first successful visualization of SSTR expression in NETs was obtained thanks to ^123^I-tyr-3-octreotide. However, due to the short half-life of ^123^I (13.2 h) and its high cost, this tracer was soon replaced by ^111^In-DTPA-octreotide, which is still commercially available (*Octreoscan,* distributed by Curium US LCC). The presence of octreotide allows this tracer to bind mainly to sst2 and sst5, and the presence of ^111^In gives it a half-life of 2.8 days. [2] [https://www.accessdata.fda.gov/drugsatfda_docs/label/2021/020314S014lbl.pdf accessed on 13 July 2022].

Another alternative for SPECT imaging of NETs is represented by ^99m^Tc-EDDA/HYNIC-TOC. This tracer has a high affinity for sst2 and a half-life of 6.02 h due to the presence of ^99m^Tc. Even this SPECT tracer is commercially available in a lyophilized form (*Tektrotyd*, distributed by ROTOP Pharma GmbH). [https://file.wuxuwang.com/hma/DE_H_3726_001_FinalSPC.pdf accessed on 13 July 2022].

Nowadays, PET investigations are increasingly arising in the nuclear medicine field, and this involves NETs’ analysis too. Therefore radioisotopes such as ^68^Ga and ^64^Cu have been used even for the radiolabeling of the SSTR’s agonists DOTATOC, DOTATATE, and DOTANOC [3]. The related tracers all show a high affinity for sst2, whereas derivatives radiolabeled with ^64^Cu have a longer half-life (12.7 h) and a lower positron range (thus an improved spatial resolution) compared to ^68^Ga ones. [https://go.drugbank.com/drugs/DB15494; https://go.drugbank.com/drugs/DB13925 accessed on 13 July 2022].

DOTATOC is commercially available as a lyophilized kit (*Somakit TOC*, distributed by Advanced Accelerator Applications), whereas DOTATATE and DOTANOC are not. Moreover, an injectable solution of ^64^Cu-DOTATATE is commercially available only in the USA (*Detecnet*, distributed by Curium US LCC) [https://go.drugbank.com/drugs/DB15494; https://go.drugbank.com/drugs/DB15873, accessed on 13 July 2022].

The labeling of ^64^Cu was even tested on the somatostatin analog SARTATE (octreotate with MeCOSar as chelant). SARTATE can bind even ^67^Cu, making this somatostatin analog suitable for theragnostic purposes. Indeed, ^64^Cu/^67^Cu-SARTATE combines the imaging properties of ^64^Cu with the therapeutic ones of ^67^Cu. Despite its high potential, studies on this new theragnostic agent are still in the preclinical stage [4,5] or limited to a small number of patients [6].

Other PET radioisotopes have been tested for NETs analysis, such as ^11^C or ^18^F, but they have shown a number of limitations [3].

Recently, a large development has been reported even for the treatment of NETs with peptide receptor radionuclide therapy (PRRT) [2]. The imaging-based diagnosis here is associated with molecular-targeted therapy, giving birth to the so-called theragnostic, which paves the way to personalized therapy, reducing the side effects associated with the treatment and maximizing the therapeutic efficacy [2].

Early clinical trials of PRRT tested octreotide radiolabeled with high doses of ^111^In for therapy. ^111^In, indeed, besides gamma particles, also emits Auger electrons with a medium-to-short tissue penetration, thus making it a suitable radionuclide for large tumor treatments. However, partial remission of the tumor mass was seen only exceptionally, and ^111^In was soon replaced in therapy by beta-emitters ^90^Y and ^177^Lu [7,8].

^90^Y-DOTATOC was first administered in patients affected by NETs in 1996, whereas ^177^Lu-DOTATATE was introduced in 2000 and received FDA approval as Luthathera in January 2018, becoming the first radiopharmaceutical approved for the therapy of gastroenteropancreatic neuroendocrine tumors (GEP-NETs) [8].

In the present systematic review, we aimed to analyze papers comparing head-to-head radiolabeled peptides for SPECT and PET imaging of NET in order to answer the following question: “Is there still a role for SPECT agents in the management (from diagnosis to therapy) of NETs in the PET era?”

## 2. Materials and Methods

This review was conducted using the Preferred Reporting Items for the Systematic Review and Meta-Analysis (PRISMA) approach. A comprehensive literature search was separately performed by G.P. and L.E in two steps. Initially, the three databases, PubMed, Web of Science, and Scopus, were searched. The following words were used to search the three databases: “68Ga-DOTATOC” and “68Ga-DOTATATE,”; “68Ga-DOTATOC” and “68Ga-DOTANOC,”; “68Ga-DOTATOC” and “99mTc-EDDA/HYNIC-TOC,”; “68Ga-DOTATOC” and “64Cu-DOTATATE,”; “68Ga-DOTATOC” and “111In-DTPA-octreotide,”; “68Ga-DOTATATE” and “68Ga-DOTANOC,”; “68Ga-DOTATATE” and “99mTc-EDDA/HYNIC-TOC,”; “68Ga-DOTATATE” and “64Cu-DOTATATE,”; “68Ga-DOTATATE” and “111In-DTPA-octreotide,”; “68Ga-DOTANOC” and “99mTc-EDDA/HYNIC-TOC,”; “68Ga-DOTANOC” and “64Cu-DOTATATE,”; “68Ga-DOTANOC” and “111In-DTPA-octreotide,”; “99mTc-EDDA/HYNIC-TOC” and “64Cu-DOTATATE,”; “99mTc-EDDA/HYNIC-TOC” and “111In-DTPA-octreotide,”; “64Cu-DOTATATE” and “111In-DTPA-octreotide,”; “68Ga TOC” and “68Ga TATE,”; “68Ga TOC” and “68Ga NOC,”; “68Ga TOC” and “99mTc TOC,”; “68Ga TOC” and “64Cu TATE,”; “68Ga TOC” and “111In octreotide,”; “68Ga TATE” and “68Ga NOC,”; “68Ga TATE” and “99mTc TOC,”; “68Ga TATE” and “64Cu TATE,”; “68Ga TATE” and “111In octreotide,”; “68Ga NOC” and “99mTc TOC,”; “68Ga NOC” and “64Cu TATE,”; “68Ga NOC” and “111In octreotide,”; “99mTc TOC” and “64Cu TATE,”; “99mTc TOC” and “111In octreotide,”; “64Cu TATE” and “111In octreotide”. No filters were applied.

Subsequently, the three databases were searched again with the following words: “Somatostatin receptor imaging”, “Somatostatin receptor imaging” and “Functional”, “Somatostatin receptor imaging” and “SPECT”, “Somatostatin receptor imaging” and “PET”. For this second round, we filtered only papers based on comparative studies.

Among the collected papers were selected the ones that meet these criteria: (1) original articles in the English language; (2) clinical application of the radiopharmaceutical agents in NETs, and (3) head-to-head comparative studies of SPECT and/or PET radiotracers in NETs imaging. Conversely, editorials, letters to the editor, reviews, pictorial essays, clinical cases, or opinions were excluded.

After the recovery of the PDF files, a new search across the reference lists in the selected studies was conducted by G.P. and L.E.

The quality of clinical papers was assessed with a modified version of the Critical Appraisal Skills Program (CASP) checklist for diagnostic studies [https://casp-uk.b-cdn.net/wp-content/uploads/2018/03/CASP-Diagnostic-Checklist-2018_fillable_form.pdf] (access on 26 July 2022). This critical appraisal was done by two reviewers (G.P. and L.E.), and any divergence in opinion was resolved by discussion with a third author (D.C.).

## 3. Results and Discussion

### 3.1. Literature Search Analysis

**Figure 1:**
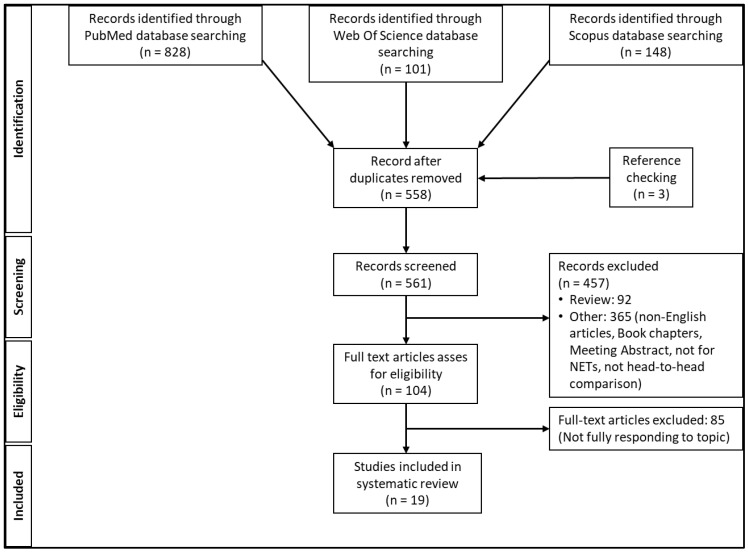
Scheme of record selection.

A total of 1077 articles were found. All duplicates were removed, leaving 558 records. Then, all reviews and all articles not entirely consistent with the inclusion criteria were excluded. The full texts of 104 articles were assessed for eligibility, and a further three articles emerged upon checking the reference lists. Finally, 19 articles were included (Figure 1)**.** The quality of the selected articles, based on the CAPS for diagnostic studies, is reported in Supplementary Table S1.

As illustrated in the supplementary material, in many cases, the studies have not included a standard of reference, or different types of analyses were used (i.e., lesion-based, region-based, or patient-based), thus rendering difficult the comparison between or among the radiopharmaceutical agents. Moreover, in many cases, the impact of the imaging on the selected population was not clearly stated.

Table 1 reports the main characteristics of 19 selected articles.

Three papers aimed to assess the comparison between ^111^In-DTPA-Octreotide and ^99m^Tc-EDDA/HYNIC-TOC [9,10,13]. In the study by Bangard et al. [9], the authors compared nine patients who underwent a scintigraph examination with both tracers and described different biokinetics between them. The uptake of ^99m^Tc-EDDA-HYNIC-TOC was lower in the spleen and kidney than ^111^In-DTPA-Octreotide. However, lesion-based analysis, ^111^In-DTPA-Octreotide, detected more lesions, mainly in the liver, while ^99m^Tc-EDDA-HYNIC-TOC identified more abdominal lesions. Conversely, in a head-to-head comparison, Decristoforo et al. [10] demonstrated, in 10 patients, that ^99m^Tc-EDDA-HYNIC-TOC was simpler to produce and more detectable of lesions than ^111^In-DTPA-Octreotide, thus, opening the way for new alternative SPECT agents for the NET detection. Three years later, Gabriel et al. [13] concluded that ^99m^Tc-EDDA-HYNIC-TOC scintigraphy is more performant than ^111^In-DTPA-Octreotide, mainly if the acquisition is made by an early and late acquisition (after 1–2 h from the tracer injection), in order to improve the tumor/background ratio.

Eleven out of 19 papers, including 498 patients, focused on the comparison between ^111^In-DTPA-Octreotide SPECT or SPECT/CT and ^68^Ga-DOTATOC PET or PET/CT. [11,12,14,15,16,17,18,19,20,22,26] Hofmann et al. [11] enrolled eight patients with metastatic carcinoid who underwent ^68^Ga-DOTATOC SPECT, CT, MRI, and ^111^In-DTPA-Octreotide, showing the power of PET tracer in detecting the lesions (100% vs. 85%, respectively for ^68^Ga-DOTATOC and ^111^In-DTPA-Octreotide). A similar and limited experience was reported by Kowalski et al. in four patients [12]. In this small patient population, the researchers found that PET was able to better detect small lesions with low-density SSTR expression. The studies by Buchmann et al. [14], Gabriel et al. [15], Srirajaskanthan et al. [17], Krausz et al. [18], Hofman et al. [19] demonstrated that ^68^Ga-DOTATOC/TATE PET/CT was able to detect more NET lesions than ^111^In-DTPA-Octreotide SPECT or SPECT/CT during patient-based and lesion-based analysis. In particular, ^68^Ga-DOTATOC/TATE PET/CT was able to better define the extension of metastatic disease in the liver, skeleton, and thoracic/abdominal lymph nodes. Moreover, based on the study by Krausz et al. [18], primary NET in the pancreas was more often detected by ^68^Ga-DOTATOC/TATE PET/CT than ^111^In-DTPA-Octreotide SPECT/CT, thus increasing its performance also in primary tumors and not only for metastatic disease. Additionally, in the papers by Buchmann et al. [14], Gabriel et al. [15], Srirajaskanthan et al. [17], and Hofman et al. [19], ^68^Ga-DOTATOC/TATE PET/CT was able to improve the clinical management in comparison with ^111^In-DTPA-Octreotide SPECT/CT. Indeed, based on the study by Buchmann et al. [14], surgical intervention was extended in seven patients owing to PET findings. Similarly, PET was able to change the therapeutic approach from a surgical to a systemic one after identifying more distant NET lesions (12/51 patients; 24%), in accordance with Gabriel et al. [15]. In the study by Srirajaskanthan et al. [17], the change of management with PET imaging was reported in 36/51 (70.6%) patients, mainly by providing the opportunity to undergo PRRT with ^90^Y/^177^Lu-DOTATATE/TOC. Finally, Hofman et al. [19] reported that PET imaging had a high management impact in 28/58 (47%) patients. Indeed, ^68^Ga-DOTATOC/TATE PET/CT increased the number of lesions detected; thus, many patients received systemic therapy rather than undergoing surgery.

Sadowski et al. [22] assessed the comparison between ^68^Ga-DOTATATE PET/CT and ^111^In-DTPA-Octreotide SPECT/CT in a cohort of patients affected by MEN1, demonstrating that ^68^Ga-DOTATATE PET/CT is more sensitive in detecting MEN1 lesions than SPECT imaging and it could also alter the management; therefore the authors strongly recommend to introduce this imaging modality in the diagnostic flow-chart of patients affected by MEN1 syndrome.

Finally, in the study by Van Binnebeek et al. [20] and Hope et al. [26] appeared, the term “tumor burden” relative to the extension of SSTR-positive disease. In both the studies, ^68^Ga-DOTATOC or ^68^Ga-DOTATATE was superior to SPECT imaging with ^111^In in assessing the tumor burden, mainly for the identification of small lesions detected by the PET scanner rather than by the SPECT one.

The articles focused on the comparison between ^68^Ga-DOTATOC/^68^Ga-DOTATATE and ^99m^Tc-EDDA/HYNIC-TOC were 2 [23,25]. In the study by Madrzak et al. [23], 24 patients underwent both images with PET/CT and SPECT/CT. The authors found that ^68^Ga-DOTATOC PET/CT altered the treatment procedures in only 8.4% of patients (2 persons). However, due to the limited patient enrolment, the authors suggested additional studies to confirm this assumption. Therefore, one year later, Kunikowska et al. [25] enrolled 68 patients showing the advantages of ^68^Ga-DOTATATE PET/CT over ^99m^Tc-EDDA/HYNIC-TOC in detecting NET lesions and underlined that ^68^Ga-DOTATATE PET/CT was able to change the clinical decision in one-third of patients.

Two articles (n = 64 patients) [24,27] assessed the comparison between ^64^Cu-DOTATATE and ^68^Ga-DOTATOC/TATE. Johnbeck et al. [24] enrolled 59 patients who underwent PET imaging with both tracers within 1 week. Through an intra-patient analysis, it emerged that PET images were concordant in 37 patients and discordant in 22 patients. Among this later subset of patients, most additional lesions were found by ^64^Cu-DOTATATE vs. ^68^Ga-DOTATOC/TATE (14 vs. eight patients, respectively). Although ^64^Cu-DOTATATE seems more performant in this study, in a very recent pilot analysis performed on five patients, Jha et al. [27] concluded that the data currently available was not conclusive about the superiority of one over the other.

Finally, the residual paper was a comparative analysis between ^111^In-DTPA-Octreotide and ^64^Cu-DOTATATE [21]. This was a large experience in 112 patients demonstrating that, similar to ^68^Ga radiolabeled peptides, ^64^Cu-DOTATATE PET is superior to ^111^In-DTPA-Octreotide SPECT.

### 3.2. Comparative Perfomances

For SPECT radiopharmaceuticals, the rate of true positive was 63.7% and 58.5%, respectively, for ^111^In-DTPA-Octreotide and ^99m^Tc-EDDA/HYNIC-TOC as expressed in studies by Gabriel et al. [13] Srirajaskanthan et al. [17], Krausz et al. [18], Pfeifer et al. [21] and Madrzak et al. [23] and illustrated in Table 2.

Conversely, for PET radiopharmaceuticals, the rate of true positivity was 78.4% and 82.4%, respectively, for ^68^Ga-DOTATOC and ^64^Cu-DOTATATE., as demonstrated by Gabriel et al. [15], Srirajaskanthan et al. [17], Krausz et al. [18], Madrzak et al. [23], and Johnbeck et al. [24] (Table 2). As clearly shown in Table 2, the number of patients with false negative results was higher for SPECT radiopharmaceuticals rather than for PET ones.

Indeed, as is visible from Table 3, the sensitivity was, as expected, higher for PET radiopharmaceuticals either with ^68^Ga and ^64^Cu than for SPECT agents.

However, by comparing ^111^In-DTPA-Octreotide and ^99m^Tc-EDDA/HYNIC-TOC, the latter has a higher sensitivity, although a lower specificity. Until now, only limited data are available on the comparison between ^68^Ga-DOTATOC/TATE and ^64^Cu-DOTATATE. The study by Johnbeck et al. [24] found a slightly higher sensitivity and specificity for ^64^Cu-DOTATATE when compared to ^68^Ga-DOTATOC. However, the data are still limited for final evidence.

For lesion-based and site-based analyses, radiopharmaceuticals for PET imaging were more performant than SPECT agents in identifying the number and the presence of lesions in the musculoskeletal system, bone, liver, and lymph nodes (mainly in the abdominal region).

The paper by Mussig et al. [16] analyzed the association between the expression of sst2 and the uptake of ^68^Ga-DOTATOC and ^111^In-DTPA-Octreotide. The authors found that a positive scan with both the tracers was associated with a high expression of sst2; however, tumors without immunohistochemical sst2 expression could show ^68^Ga-DOTATOC tracer uptake, probably due to the expression of sst3 or sst5, or simply because of the tumor heterogeneity.

### 3.3. The Theragnostic Role of Radiopharmaceuticals for NET

The theragnostic role of the abovementioned radiopharmaceuticals in the different settings of disease (detection, staging, status of SSTR, and follow-up) has scarcely been reported in comparative studies.

From a careful analysis of the available data, 56 (7.8%) patients were enrolled for the detection of NET, 120 (16.9%) for staging, and 535 (75.3%) for the assessment of SSTR expression and follow-up. As expected, follow-ups for the evaluation of SSTR expression were the most common indication in many selected studies. Higher diagnostic performances have been reported for the assessment of SSTR status in the follow-up settings for PET tracers as compared to SPECT tracers, for patient-based analysis but also regional- and lesion-based ones. After different previous treatments, the assessment of SSTR expression is essential in planning PRRT, and the PET tracer results were more accurate in these settings at any level of analysis. However, no information has been found about PET and SPECT tracers in monitoring the response to PRRT in NETs, although it would be an interesting and important topic from a diversified point of view.

## 4. Discussion and Conclusions

Scintigraphy with radiolabeled SSTR has gained widespread acceptance as the imaging method of choice in NET patients, showing high sensitivity and good specificity, as emerged from the previous study and from this systematic review. From planar imaging with ^111^In-DTPA-Octreotide to SPECT with ^99m^Tc-HYNIC/EDDA-TOC, a gain in terms of detection has been obtained. However, due to the limited spatial resolution of SPECT imaging, PET tracers radiolabeled with either ^64^Cu or ^68^Ga have been introduced in clinical practice, thus increasing the detection of NET lesions.

From the present comparative review, it emerged that for patient-based analysis, the rate of true positive and the diagnostic performance is as expected, higher for PET tracers when compared to SPECT tracers, mainly when ^111^In-DTPA-Octreotide was compared to ^68^Ga-DOTATOC/TATE/NOC. Moreover, when analyzing the available data for lesion- and region-based analyses, we found that PET radiopharmaceutical agents were more performant in detecting bone, lymph nodes, and liver metastases than SPECT agents. This advantage was mainly due to the PET scanner technology rather than the radiopharmaceutical itself. It would be interesting to understand if new technological achievements in SPECT technology, such as solid-state detectors and 360° detector coverage, could fill this gap. Indeed, PET tomographic images can significantly improve the detection of deep lesions or visceral metastases when compared with planar or SPECT images.

By a comparative analysis between ^64^Cu-DOTATATE and ^68^Ga-DOTATOC emerged that the performances were quite similar; however, the number of true positive lesions was slightly higher for ^64^Cu-DOTATATE than ^68^Ga-DOTATOC (33 vs. 7), as reported by Johnbeck et al. [24] The affinity for SSTR was quite similar for all the imaging agents, particularly for those used in PET, as recently reported by some authors [28,29,30]. Nevertheless, the intrinsic physical characteristics of radioisotopes can have an important effect on lesion detectability. Indeed, ^64^Cu has a shorter positron range than ^68^Ga, thus possibly improving the detection rate of small lesions. Moreover, the radiation burden is different between ^64^Cu and ^68^Ga. Similarly, ^99m^Tc has the advantage of a lower radiation dose than ^111^In. This latter physical characteristic can be translated into the advantageous use of ^99m^Tc for repeated investigations, for example, in monitoring the response to PRRT or in children. However, to date, no information about the cost-saving, other than the radioprotection information, is available for ^99m^Tc-EDDA/HYNIC-TOC SPECT in comparison to ^68^Ga-DOTATOC/TATE PET. Conversely, Schreiter et al. [31] found that ^68^Ga-DOTATOC PET/CT was considerably cheaper than ^111^In-DTPA-octreotide with respect to both material and personnel costs. Therefore, additional cost analyses are welcome also for the other agents.

It should be noted, however, that comparisons between PET and SPECT agents were made considering different acquisition protocols. Table 4 reports some of the pros and cons of SPECT and PET imaging for detecting NETs.

The question that arises from the above considerations is, “Can the improved detection rate affect clinical management?”

In NETs, changes in treatment strategy are nearly always based on clinical or imaging-based signs of progression. Thus, high performance in the detection of any new lesions is of great value in patients affected by these rare diseases. For example, the additional evidence of bone metastases can have either an important effect on the therapeutic intervention or a prognostic implication because unknown distant metastases are considered a negative prognostic factor, possibly requiring a more aggressive treatment regimen [32,33]. Based on the available data, the inclusion of PET imaging in clinical practice impacted the change of management from 3.7% to 70.6% (Table S2) of patients. Therefore, PET imaging should be preferred to SPECT imaging when available. 

From a careful analysis of the selected studies, no comparative data were available about the role of PET and SPECT imaging in monitoring the response to PRRT. The recent introduction of PRRT in clinical practice (Netter 1 trial) and the opportunity of monitoring the response to therapy, both in the interim and at the end, is essential for testing the efficacy of therapy. To date, some studies have been published about the role of ^68^Ga-DOTATOC/TATE/NOC in monitoring the response to PRRT in comparison to morphological criteria without reporting conclusions [34,35]. Indeed, functional imaging is not yet accepted as a substitute for morphological imaging as a means to assess tumor response to treatment [36]. However, the opportunity to use both SPECT (especially with new scanners) and PET tracers during and after PRRT would also be an advantage in the case of retreatment or an early treatment interruption. Future studies should be conducted to test these hypotheses.

In this systematic review, we focused our attention on SPECT/PET radiotracers based on SSTR analogs, though theoretically, other PET tracers can be used for NETs imaging.

One alternative is represented by ^11^C-hydroxytriptophan (^11^C-5-HTP), a serotonin precursor that allows the evaluation of the serotonin pathway, which is one of the active metabolic pathways in NETs [37]. This tracer has a high sensitivity, especially for pancreatic NETS, but its use is limited by the half-life of the radionuclide (20 min), which requires the presence of an on-site cyclotron [3,37].

^18^F-DOPA (^18^F-L-dihydroxyphenylalanine) is another PET tracer that finds a high application in NETs [37]. Indeed, NETs cells can often take up decarboxylate monoamine precursors, such as DOPA. ^18^F-DOPA seems to be useful for imaging well-differentiated midgut tumors, though they often overexpress sst2 [38].

In a recent meta-analysis of head-to-head comparative studies emerged that at patient-based and region-based analysis, ^68^Ga-DOTA-peptides performed better than ^18^F-DOPA PET in detecting intestinal NETs, but at lesion-based analysis, ^18^F-DOPA PET was more accurate [38].

Another alternative to peptide analogs is represented by ^18^F-FDG (^18^F-fluorodeoxyglucose), which is recently becoming the PET tracer of choice in many cancer forms. ^18^F-FDG exploits cancer cells’ preferential utilization of aerobic glycolysis. Similarly, to glucose, it enters cells via glucose transporters GLUT-1 and GLUT-3, but it doesn’t follow the same metabolic pathway of glucose due to a lack of an oxygen atom in its C2 position. Thus, it accumulates in cells proportional to their glucose consumption. [37] However, for many years it was not used in NETs due to its low sensitivity in the detection of these tumors, but more recently, the utility of FDG-PET scans has been reassessed [1,3]. NETs with poor differentiation, a high grade, and rapid proliferation have a decreased expression of SSTR expression; thus, scans with peptide analogs may be negative, while ^18^F-FDG imaging may be positive [1,39]. Liu et al. [39] analyzed 30 studies focused on ^68^Ga-radiolabelled agonist SSTR and FDG PET/CT in NET patients. From the meta-analysis emerged that ^18^F-FDG PET/CT has the lowest sensitivity in detecting NET lesions. However, it has a complementary role in the case of moderately or scarcely differentiated NETs.

The abovementioned radiopharmaceuticals have shown promising results, but these substances do not provide any theragnostic options, unlike somatostatin analogs.

Lastly, new interest is increasing in the use of SSTR antagonists. Compared to agonists, they showed better pharmacokinetics and image contrast, a higher tumor uptake, and a better residence time [37], [40]. Among them, ^68^Ga-NOGADA-JR11 (or ^68^Ga-OPS202) and ^68^Ga-DOTA-JR11 have also demonstrated advantages for potential theragnostic application [40,41,42]. In particular, the study by Zhu et al. [40] showed that in 12 patients undergoing imaging with both radiopharmaceutical agents on two consecutive days, ^68^Ga-DOTA-JR11 outperformed better that ^68^Ga-DOTATATE in detecting liver metastases, while ^68^Ga-DOTATATE was better for the identification of bone lesions. However, to date, little clinical evidence is still available.

The present systematic review has limitations. The limited number of studies comparing ^64^Cu-DOTATATE vs. ^68^Ga-DOTATATE. Moreover, in the study by Pfeifer et al. [21], ^64^Cu-DOTATATE PET/CT was compared with ^68^Ga-DOTATOC PET, therefore by using a hybrid vs. non-hybrid system, thus reducing the detection power of the second imaging modality. In the study by Buchman et al. [14], the authors reported that the region-based analysis could have overestimated the sensitivity of ^111^In-DTPA-octreotide. Few data about the standard of reference, as also emerged by the CAPS evaluation; indeed, it missed 10/19 (53%) of papers, thus reducing the opportunity to perform an adequate comparison in terms of diagnostic performances.

In conclusion, PET imaging, as expected, is more suitable for the identification of NET. Indeed, they demonstrated a higher true positive rate than SPECT imaging. However, the availability of new SPECT scanners, more favorable radioprotection and synthetical characteristics (mainly for ^99m^Tc), and the consolidated experiences for conventional scintigraphy examination should be considered in the diagnostic and therapeutic path, also for health equity.

## Figures and Tables

**Table 1:** Characteristics of the selected studies.

	Author, Ref	Year of Pub	Country	N pts	Comparative RF	SOR	Interpretation	Outcome
1	Bangard et al. [9]	2000	Germany	9	^111^In-DTPA-OC vs. ^99m^Tc-EDDA/HYNIC-TOC	None	Visual ans semiquantitative analysis	Both tracers outperform similarly for the detection of tumors, although ^99m^Tc-EDDA/HYNIC-TOC detects more abdominal lesions and ^111^In-DTPA-OC more liver metastases
2	Decristoforo et al. [10]	2000	Austria	10	^111^In-DTPA-Octreotide/^111^In-DTPA-TOC vs. ^99m^Tc-EDDA/HYNIC-TOC	None	Visual ans semiquantitative analysis	Both tracers outperform similarly for the detection of tumor
3	Hofmann et al. [11]	2001	Germany	8	^111^In-DTPA-Octreotide vs. ^68^Ga-DOTATOC	None	Visual analysis by three observers	^68^Ga-DOTATOC outperformed ^111^In-DTPA-Octretide detecting more lesions
4	Kowalski et al. [12]	2003	Germany	4	^111^In-DTPA-Octreotide vs. ^68^Ga-DOTATOC	None	Visual analysis	^68^Ga-DOTATOC outperformed ^111^In-DTPA-Octretide detecting more lesions
5	Gabriel et al. [13]	2003	Austria	41	^111^In-DTPA-Octreotide vs. ^99m^Tc-EDDA/HYNIC-TOC.	Imaging	Visual analysis by two observers	^99m^Tc- EDDA/HYNIC- TOC outperformed ^111^In-DTPA-Octretide detecting more lesions
6	Buchmann et al. [14]	2007	Germany	27	^111^In-DTPA-Octreotide vs. ^68^Ga-DOTATOC	Histology and imaging	Visual analysis by two observers	^68^Ga-DOTATOC outperformed ^111^In-DTPA-Octretide detecting more lesions and changing the management
7	Gabriel et al. [15]	2007	Austria	84	^111^In-DTPA-Octreotide vs. ^68^Ga-DOTATOC	Histology and imaging	Visual analysis by two observers (third in discordant case)	^68^Ga-DOTATOC outperformed ^111^In-DTPA-Octretide detecting more lesions and changing the management
8	Mussig K et al. [16]	2010	USA	36	^111^In-DTPA-Octreotide vs. ^68^Ga-DOTATOC	Histology	Visual analysis by two observers	^111^In-DTPA-Octreotide and ^68^Ga-DOTATOC both correlate with SSTR expression
9	Srirajaskanthan et al. [17]	2010	UK	51	^111^In-DTPA-Octreotide vs. ^68^Ga-DOTATATE	None	Visual analysis	^68^Ga-DOTATATE outperformed ^111^In-DTPA-Octretide detecting more lesions and changing the management
10	Krausz et al. [18]	2011	Israel	19	^111^In-DTPA-Octreotide vs. ^68^Ga-DOTANOC	None	Visual analysis	^68^Ga-DOTANOC outperformed ^111^In-DTPA-Octretide detecting more lesions and changing the management
11	Hofman et al. [19]	2012	Australia	40	^111^In-DTPA-Octretide vs. ^68^Ga-DOTATATE	None	Visual analysis	^68^Ga-DOTATATE outperformed ^111^In-DTPA-Octretide detecting more lesions
12	Van Binnebeek et al. [20]	2016	Belgium	53	^111^In-DTPA-Octreotide vs. ^68^Ga-DOTATOC	Imaging	Visual analysis	^68^Ga-DOTATOC outperformed ^111^In-DTPA-Octretide detecting more lesions
13	Pfeifer et al. [21]	2015	Denmark	112	^111^In-DTPA-Octretide vs. ^64^Cu-DOTATATE	Histology and imaging	Visual analysis by two observers	^64^Cu-DOTATATE outperformed ^111^In-DTPA-Octretide detecting more lesions
14	Sadowski et al. [22]	2015	Austria	26	^111^In-DTPA-Octreotide vs. ^68^Ga-DOTATOC	Histology and imaging	Visual analysis	^68^Ga-DOTATOC outperformed ^111^In-DTPA-Octretide detecting more lesions without changing the management
15	Madrzak et al. [23]	2016	Poland	24	^99m^Tc-EDDA/HYNIC-TOC vs. ^68^Ga-DOTATOC/TATE	Not clear	No data	^68^Ga-DOTATOC outperformed ^99m^Tc-EDDA/HYNIC-TOC by detecting more lesions and by changing the management
16	Johnbeck et al. [24]	2017	Denmark	59	^64^Cu-DOTATATE vs. ^68^Ga-DOTATOC	Clinical follow-up	Visual analysis by one observer	^64^Cu-DOTATATE outperformed ^68^Ga-DOTATOC detecting more lesions
17	Kunikowska et al. [25]	2017	Poland	68	^99m^Tc-HYNIC-TOC vs. ^68^Ga-DOTATATE	Imaging	Visual analysis	^68^Ga-DOTATATE outperformed ^99^Tc-HYNIC-TOC detecting more lesions, and by changing the management
18	Hope et al. [26]	2019	USA	150	^111^In-DTPA-Octreotide vs. ^68^Ga-DOTATOC	None	Visual and semiquantitative analysis	^68^Ga-DOTATOC outperformed ^111^In-DTPA-Octreotide in higher Krenning score lesions (mainly for size < 2 cm
19	Jha et al. [27]	2022	USA	5	^64^Cu-DOTATATE vs. ^68^Ga-DOTATATE	None	Visual analysis	Both ^64^Cu-DOTATATE and ^68^Ga-DOTATATE can be interchangeably

SOR = standard of reference; SSA: somatostatin analogs

**Table 2:** Patient-based performances of the diverse radiopharmaceutical agents.

	Author, Ref	N pts	^111^In-DTPA Octreotide				^99m^Tc-EDDA/HYNIC-TOC				^68^Ga-DOTATATE/NOC/TOC				^64^Cu-DOTATATE			
			TP	TN	FP	FN	TP	TN	FP	FN	TP	TN	FP	FN	TP	TN	FP	FN
1	Gabriel et al. [13]	41	21	4	1	15	27	2	3	9	-	-	-	-	-	-	-	-
2	Gabriel et al. [15]	84	37	12	1	34	**	**	**	**	69	12	1	2	-	-	-	-
3	Srirajaskanthan et al. [17]	51	15	3	1	32	-	-	-	-	42	3	1	5	-	-	-	-
4	Krausz et al. [18]	19	19	0	0	0	-	-	-	-	19	0	0	0	-	-	-	-
5	Pfeifer et al. [21]	112	87	12	0	13	-	-	-	-	-	-	-	-	97	12	0	3
6	Madrzak et al. [23]	24	-	-	-	-	11	11	1	1	12	12	0	0	-	-	-	-
7	Johnbeck et al. * [24]	59	-	-	-	-	-	-	-	-	43	9	1	5	43	14	1	0

* Follow-up was unverified in 1 patient; ** Both ^111^In-DTPA-Octreotide and ^99m^Tc-HYNIC-TOC for SPECT imaging.

**Table 3:** Diagnostic accuracies of some selected papers.

	Author, Ref	^111^In-DTPA-Octreotide			^99m^Tc-EDDA/HYNIC-TOC			^68^Ga-DOTATATE/NOC/TOC			^64^Cu-DOTATATE		
		Sens (95% CI)	Spec (95% CI)	Acc (95% CI)	Sens (95% CI)	Spec (95% CI)	Acc (95% CI)	Sens (95% CI)	Spec (95% CI)	Acc (95% CI)	Sens (95% CI)	Spec (95% CI)	Acc (95% CI)
1	Gabriel et al. [13]	**58.3** (42–73)	**80** (64–90)	**61**	**75** (58–87)	**40** (25–56)	**70.7**	-	-	-	-	-	-
2	Gabriel et al. [15]	**52.1** (41–63)	**92.3** (84–97)	**58.3**	**	**	**	**97.2** (90–99)	**92.3** (84–97)	**96.4**	-	-	-
3	Srirajaskanthan et al. [17]	**31.9** (20–46)	**75** (61–86)	**35**	-	-	-	**89.4** (77–96)	**75** (61–86)	**88**	-	-	-
4	Pfeifer et al. [21]	**87** (79–92)	**100** (96–100)	**88.4**	-	-	-	-	-	-	**97** (91–99)	**100** (96–100)	**97.3**
5	Madrzak et al. [23]	-	-	-	**91.7** (72–99)	**91.7** (72–99)	**91.7**	**100** (83–100)	**100** (83–100)	**100**	-	-	-
6	Johnbeck et al. * [24]	-	-	-	-	-	-	**89.6** (78–96)	**90** (79–96)	**89.7**	**100** (92–100)	**93** (83–98)	**98.3**

* Follow-up was unverified in 1 patient; ** Both ^111^In-DTPA-Octreotide and ^99m^Tc-Hynic-TOC for SPECT imaging.

**Table 4:** Advantages and disadvantages for PET and SPECT imaging in NET.

PROS		CONS	
SPECT	PET	SPECT	PET
High availability of gamma-camera scanners High clinical experience, mainly with ^111^In-DTPA-octreotide High availability of ^99m^Tc	Higher spatial resolution [15,17] Improved target-to-background contrast [18] Broader affinity of PET tracers for SSTR [18] High detection rate for PET tracers [15,18] Improved PK [18] Less time between tracer injection and scan acquisition [14], [18] Fast acquisition protocols [18] Lower effective dose equivalent [18] Lower unspecific radiation exposure of medical personnel [14] Possibility to quantify findings [14] Potential use for monitoring therapy response [14]	Limitation on liver metastases detection [15] 2 days protocol [14,18] Whole-body SPECT is uncomfortable for patients [14] Hardly associated with CT [18] Low availability of ^111^In-DTPA-octreotide	Limited availability of PET tracers [14] Limited availability of PET scanners

PK = pharmacokinetic, CT = computed tomography.

## Data Availability

Not applicable.

## Supplementary Materials

The following supporting information can be downloaded at: https://www.mdpi.com/article/10.3390/cimb44110373/s1, Table S1: Clinical studies assessment using Critical Appraisal Skill Progam (CASP) checklist for diagnostic studies; Table S2: Change of management with PET imaging on patients affected by the neuroendocrine tumor.
