# Supplementary material for: Head-to-Head Comparison between Peptide-Based Radiopharmaceutical for PET and SPECT in the Evaluation of Neuroendocrine Tumors: A Systematic Review
Source: Curr Issues Mol Biol. 2022 Nov 7;44(11):5516–30. doi: 10.3390/cimb44110373 (PMC9689511; doi:10.3390/cimb44110373)
Supplement: Supplementary file 1 [file cimb-44-00373-s001.zip › cimb-1877024-supplementary.pdf]

Table S1. Clinical studies assessment using Critical Appraisal Skills Programme (CASP) checklist for diagnostic studies

Questions	Initials	Ref.	[16]	[18]	[14]	[17]	[21]	[13]	[24]	[15]	[23]	[27]	[12]	[11]	[9]	[10]	[20]	[25]	[22]	[19]	[26]
1. Was there a clear question for the study to address?	*GP*		Y	Y	Y	Y	Y	Y	Y	Y	Y	CT	CT	Y	Y	Y	Y	Y	CT	CT	Y
	LE		Y	Y	Y	Y	Y	Y	Y	Y	Y	Y	CT	Y	Y	Y	Y	Y	Y	CT	Y
	AG											**Y**							**Y**		
2. Was there a comparison with an appropriate reference standard?	*GP*		N	Y	Y	Y	Y	Y	Y	Y	Y	Y	Y	Y	Y	Y	Y	Y	Y	Y	Y
	LE		N	Y	Y	Y	Y	Y	Y	Y	Y	Y	Y	Y	Y	N	Y	Y	Y	N	Y
	AG															**N**				**N**	
3. Did all patients get the diagnostic test and reference standard?	*GP*		CT	Y	Y	Y	Y	Y	Y	Y	N	Y	CT	Y	N	N	Y	Y	CT	CT	Y
	LE		Y	N	Y	N	Y	Y	Y	Y	CT	N	N	Y	N	N	CT	Y	Y	N	CT
	AG		**Y**	**N**		**N**					**CT**	**N**	**N**				**CT**		**Y**	**N**	**CT**
4. Could the results of the test have been influenced by the results of the reference standard?	*GP*		CT	CT	CT	CT	CT	CT	CT	CT	CT	CT	CT	CT	CT	CT	CT	CT	CT	CT	CT
	LE		CT	CT	CT	CT	CT	CT	CT	CT	CT	CT	CT	CT	CT	CT	CT	N	N	CT	CT
	AG																	**N**	**N**		
5. Is the disease status of the tested population clearly described?	*GP*		Y	Y	CT	CT	Y	Y	Y	Y	CT	N	CT	Y	CT	Y	Y	CT	CT	Y	N
	LE		Y	Y	Y	Y	Y	Y	Y	Y	Y	N	CT	Y	CT	Y	Y	Y	Y	Y	N
	AG				**Y**	**Y**					**Y**							**Y**	**Y**		
6. Were the methods for performing the test described in sufficient detail?	*GP*		Y	Y	Y	Y	Y	Y	Y	Y	CT	N	Y	Y	Y	Y	Y	Y	N	N	Y
	LE		Y	Y	Y	Y	Y	Y	Y	Y	Y	N	Y	Y	Y	Y	Y	Y	N	N	Y
	AG										**Y**										
7. What are the results?	*GP*		CLR	CLR	CLR	CLR	CLR	CLR	CLR	CLR	CLR	CLR	CLR	CLR	CLR	CLR	CLR	CLR	NCLR	NCLR	CLR
	LE		CLR	CLR	CLR	CLR	CLR	CLR	CLR	CLR	CLR	CLR	CLR	CLR	CLR	CLR	CLR	CLR	NCLR	NCLR	CLR
	AG																				

8. How sure are we about the results? Consequences and cost of alternatives performed?	*GP*		MED	MED	MED	HIG	HIG	HIG	HIG	HIG	HIG	LOW	LOW	LOW	MED	LOW	MED	HIG	LOW	LOW	HIG
	LE		MED	MED	MED	HIG	HIG	HIG	HIG	HIG	HIG	LOW	LOW	LOW	MED	LOW	MED	HIG	LOW	LOW	HIG
	AG																				
9. Can the results be applied to your patients/ the population of interest?	*GP*		CT	CT	CT	CT	CT	CT	CT	CT	CT	CT	CT	CT	Y	Y	CT	CT	CT	CT	CT
	LE		Y	Y	Y	Y	Y	Y	Y	Y	Y	CT	CT	CT	Y	CT	CT	Y	Y	CT	CT
	AG		**Y**	**Y**	**Y**	**Y**	**Y**	**Y**	**Y**	**Y**	**Y**					**CT**		**Y**	**Y**		
10. Can the test be applied to your patient or population of interest?	*GP*		N	CT	CT	CT	CT	CT	CT	CT	CT	CT	CT	CT	Y	Y	CT	CT	CT	CT	CT
	LE		Y	Y	Y	Y	Y	Y	Y	Y	Y	CT	CT	CT	Y	CT	CT	Y	Y	CT	CT
	AG		**Y**	**Y**	**Y**	**Y**	**Y**	**Y**	**Y**	**Y**	**Y**					**CT**		**Y**	**Y**		
11. Were all outcomes important to the individual or population considered?	*GP*		CT	Y	Y	CT	Y	Y	CT	Y	CT	CT	Y	Y	Y	Y	Y	Y	CT	Y	N
	LE		CT	CT	Y	CT	Y	Y	CT	Y	CT	CT	CT	CT	Y	CT	Y	Y	CT	CT	N
	AG			**CT**									**CT**	**CT**		**CT**				**CT**	
12. What would be the impact of using this test on your patients/population?	*GP*		DK	DK	DK	DK	DK	HIG	DK	HIG	HIG	DK	DK	DK	DK	CLD	CLD	CLD	DK	CLD	DK
	LE		CLD	DK	DK	DK	CLD	HIG	DK	HIG	HIG	DK	DK	DK	DK	CLD	CLD	CLD	DK	CLD	DK
	AG		**CLD**				**CLD**														

GP: Giulia Poletto; LE: Laura Evangelista; AG: agree; Y: yes; N: No, CT: can't tell; CLR: clear; NCLR: not clear; MED: medium; HIG: high; DK: don't know; CLD: it could

Table S2. Change of management with PET imaging in patients affected by neuroendocrine tumor

**Authors, ref**	**Number of patients with change/total study population**	**% of change management**
Buchmann et al	1/27	3.7%
Srirajaskanthan et al,	36/51	70.6%
Gabriel et al (2007),	12/84	14.3%
Madrzak et al,	2/24	8.4%
Krausz et al,	3/19	15.8%
